# Recent approach for preventing complications in upper gastrointestinal endoscopic submucosal dissection

**DOI:** 10.1002/deo2.60

**Published:** 2021-10-31

**Authors:** Waku Hatta, Tomoyuki Koike, Hiroko Abe, Yohei Ogata, Masahiro Saito, Xiaoyi Jin, Takeshi Kanno, Kaname Uno, Naoki Asano, Akira Imatani, Atsushi Masamune

**Affiliations:** ^1^ Division of Gastroenterology Tohoku University Graduate School of Medicine Miyagi Japan

**Keywords:** complications, duodenum, endoscopic submucosal dissection, esophagus, stomach

## Abstract

Although endoscopic submucosal dissection (ESD) is a minimally invasive treatment method for upper gastrointestinal (GI) tumors, patients undergoing upper GI ESD sometimes fall into a serious condition from complications. Thus, it is important to fully understand how to prevent complications when performing upper GI ESD. One of the major complications in esophageal and gastric ESD is intraoperative perforation. To prevent this complication, blind dissection should be avoided. Traction‐assisted ESD is a useful technique for maintaining good endoscopic view. This method was proven to reduce the incidence of intraoperative perforation, which would become a standard technique in esophageal and gastric ESD. In gastric ESD, delayed bleeding is the most common complication. Recently, a novel prediction model (BEST‐J score) consisting of 10 factors with four risk categories for delayed bleeding in gastric ESD was established, and a free mobile application is now available. For reducing delayed bleeding in gastric ESD, vonoprazan ≥20 mg/day is the sole reliable method in the current status. Duodenal ESD is still challenging with a much higher frequency of complications, such as perforation and delayed bleeding, than ESD in other organs. However, with the development of improved devices and techniques, the frequency of complications in duodenal ESD has been decreasing. To prevent intraoperative perforation, some ESD techniques, such as using the distal tips of the Clutch Cutter, were developed. An endoscopic mucosal defect closure technique would be mandatory for preventing delayed complications. However, several unresolved issues, including standardization of duodenal ESD, remain and further studies are demanded.

## INTRODUCTION

Endoscopic submucosal dissection (ESD) is now widely accepted as a minimally invasive treatment method for early‐stage esophageal and gastric cancers, particularly in Eastern Asian countries.^1^, ^2^, ^3^, ^4^, ^5^, ^6^ In addition, many reports have shown favorable long‐term outcomes after ESD for such tumors, irrespective of the curative status,^6^, ^7^, ^8^, ^9^, ^10^, ^11^ and risk of lymph node metastasis or recurrence in noncurative resection.^12^, ^13^, ^14^, ^15^, ^16^

Meanwhile, patients undergoing esophageal and gastric ESD sometimes fall into a serious condition from complications. Furthermore, duodenal ESD is still not standardized and appears to have a higher frequency of complications than ESD in other organs. For endoscopists performing upper gastrointestinal (GI) ESD, it is important to fully understand how to prevent complications. In this review, we focused on the complications in upper GI ESD and provide a summary of recent approach to prevent them.

## ESOPHAGEAL ESD

Esophageal ESD has become the standard technique over endoscopic mucosal resection (EMR) in the treatment of esophageal cancer because it is associated with a lower recurrence rate and better survival.^17^ As major complications in esophageal ESD, intraoperative and delayed perforation, delayed bleeding, aspiration pneumonia, and stricture have been reported (Table 1).

**TABLE 1:** Major complications and management for preventing them in esophageal endoscopic submucosal dissection (ESD)

	Frequency	Preventative method
Intraoperative perforation	1.4%–4.6%^18^	Traction‐assisted method to prevent “blind” dissection^20^, ^21^
Delayed perforation	Rare (three cases; two of them required emergency surgery)^24^, ^25^	Prevention of excessive energizing during ESD^23^
Delayed bleeding	0.0%–6.7%^3^, ^27^, ^28^, ^29^, ^30^, ^31^, ^32^, ^33^, ^34^	No established method
Aspiration pneumonia	1.6%–4.0%^3^, ^38^	Use of a tube or a mouthpiece for continuous saliva suction^39^, ^40^ Use of a continuously liquid‐sucking catheter attachment for the endoscope^38^
Stricture	0.7% (<1/2 circumferential lesion) 27.6% (1/2–3/4 circumferential lesion) 94.1% (>3/4 circumferential lesion)^43^	Steroid injection^45^, ^46^, ^47^, ^48^, ^49^, ^50^, ^51^ Oral steroid intake^53^, ^54^, ^55^, ^56^, ^58^ Endoscopic balloon dilation^44^

### Perforation

According to a database analysis of 12,899 cases, the rates of perforation in very low to very high hospital volume were 4.6%–1.4%.^18^ Over three‐fourths of circumferential resection of the esophagus^19^ and a lower hospital volume^3^, ^18^ were reported as risk factors for perforation. Furthermore, blind dissection should be avoided to prevent intraoperative perforation.^20^ In this regard, traction‐assisted ESD is useful to maintain a good endoscopic view, which can overcome some of the technical difficulty associated with ESD. According to a multicenter randomized trial, no intraoperative perforation occurred in traction‐assisted ESD using dental floss, whereas 4.3% of the cases had intraoperative perforation in conventional ESD.^21^ Nevertheless, esophageal ESD has the potential for a higher incidence of complications and, thus, it is preferable that an expert endoscopist performs this procedure.^22^

Delayed perforation might be caused by tissue necrosis and degeneration by heat denaturation in the muscularis propria due to excessive energizing during ESD.^23^ GI motility, digestive juice, and/or food may be the final trigger for delayed perforation.^24^, ^25^ However, this complication is rare in esophageal ESD; indeed, there are only a few case reports about delayed perforation in esophageal ESD.^23^, ^26^ Once delayed perforation occurs, surgical treatment is generally selected,^26^ but a case was successfully treated by temporary stent replacement.^23^

### Delayed bleeding

Delayed bleeding in esophageal ESD is considered relatively rare, and the rate of this complication was reported as 0.0%–6.7%.^3^, ^27^, ^28^, ^29^, ^30^, ^31^, ^32^, ^33^, ^34^ No studies have reported the risk factors for delayed bleeding in esophageal ESD. Although the guidelines do not recommend proton pump inhibitor (PPI) after esophageal ESD,^22^ a recent large‐scale database study revealed that vonoprazan, which is a novel oral potassium‐competitive acid blocker with strong and sustained acid‐inhibitory activity,^35^ had a tendency to reduce delayed bleeding after esophageal ESD in the middle or lower part of the esophagus.^34^ This report addressed the possible reasons as the lower clearance of refluxate by esophageal motility impairment after esophageal ESD^36^ and the necessity of strict acid suppression to control bleeding (pH > 6 is required in upper GI bleeding^37^). However, further studies are demanded for confirming the advantage of vonoprazan to prevent delayed bleeding in esophageal ESD and its cost‐effectiveness should also be evaluated.^34^

### Aspiration pneumonia

**FIGURE 1:**
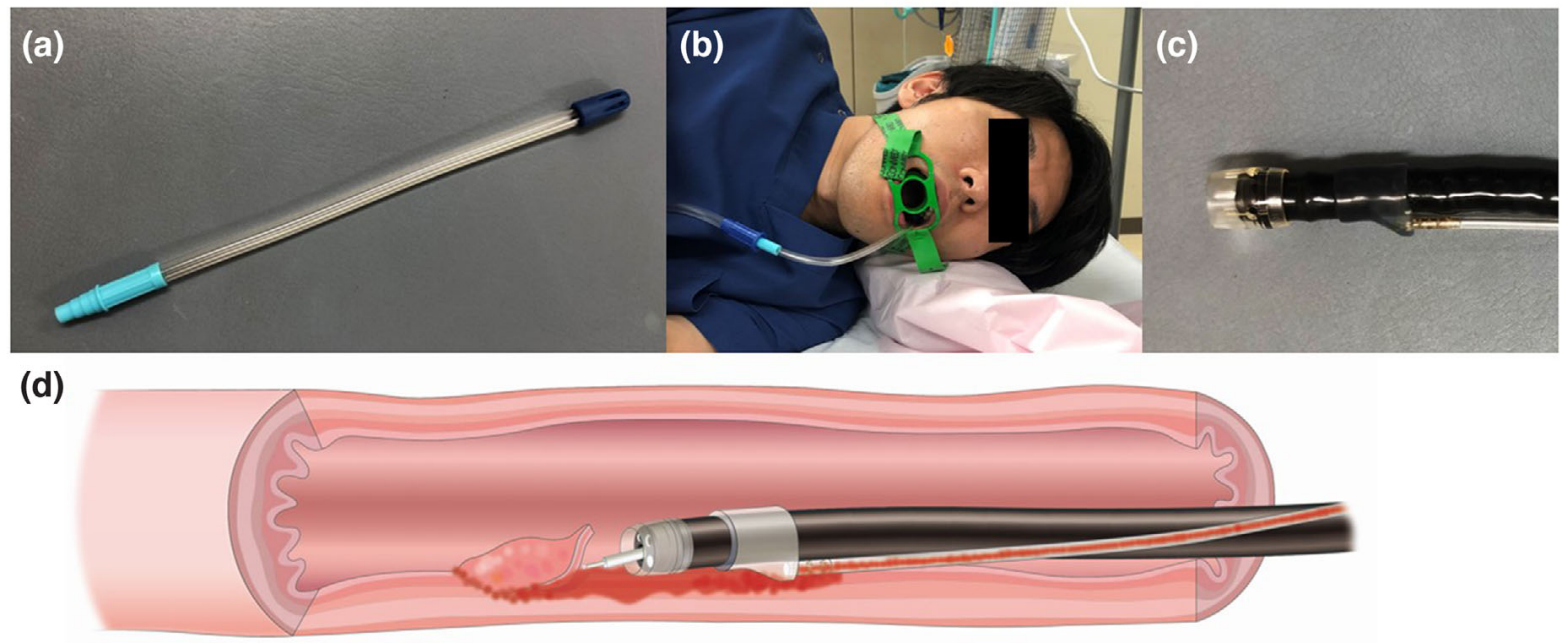
Preventative methods for aspiration pneumonia in esophageal endoscopic submucosal dissection (ESD). A tube for continuous saliva suction (a and b) and a continuously liquid‐sucking catheter attachment for the endoscope (c and d)

Aspiration pneumonia occurs in 1.6%–4.0% of esophageal ESD.^3^, ^38^ Although clinical symptoms are mild in most patients, symptoms can become serious in elderly patients. The cause of this complication is considered to be liquid reflux from the esophagus to the mouth and saliva retention in the oral cavity.^38^, ^39^ To prevent pneumonia, some methods have been developed. One is continuous saliva suction using a tube^39^ (Figure 1a,b) or mouthpiece.^40^ The other is a continuously liquid‐sucking catheter attachment for the endoscope to reduce the volume of liquid reflux to the mouth (Figure 1c,d).^38^ A randomized controlled trial revealed that the use of this device in esophageal ESD reduced the volume of liquid reflux to the mouth and contributed to the decreased incidence of aspiration pneumonia on computed tomography scan.^38^ Since the age peak has risen in esophageal cancer in Japan,^41^ the issue of aspiration pneumonia in esophageal ESD will be more important in the near future. These devices might help reduce this complication.

### Stricture

The circumferential range for resection is a well‐known risk factor for a stricture after esophageal ESD.^42^, ^43^ According to a previous study, the incidence of strictures after esophageal ESD was 0.7% in lesions with a circumferential range of <1/2, 27.6% for >1/2, and 94.1% for >3/4.^43^ A stricture remarkably decreases the quality of life of the patients. Thus, a preventative method is recommended for lesions extending >1/2 of the esophageal circumference (expected mucosal defect is ≥2/3 of the esophageal circumference) and mandatory in those >3/4 (expected mucosal defect is ≥5/6 of the esophageal circumference).

Several methods have been proposed for preventing stricture after esophageal ESD. Prophylactic endoscopic balloon dilation (EBD) decreases the incidence of a stricture after ESD.^44^ Local steroid injection is the most frequent method for preventing a stricture, and several studies have demonstrated its efficacy for reducing strictures after ESD.^45^, ^46^, ^47^ In the guidelines,^22^ this method is recommended after ESD with mucosal defects affecting ≥3/4 of the esophageal circumference. However, the details of local steroid injection have not been standardized. Triamcinolone was mostly used,^45^, ^46^, ^48^, ^49^, ^50^ but some studies used dexamethasone as well.^47^, ^51^ The number and timing of steroid injection varied depending on the study, and the dose of steroid also varied (e.g., the dose of triamcinolone injection immediately after ESD varied from 40 to 100 mg among studies with single triamcinolone injection^46^, ^48^, ^50^). Thus, standardization of the details of local steroid injection is needed. Furthermore, local steroid injection alone may not be sufficient for preventing stricture after 5/6 to entire circumferential resection.^48^, ^49^, ^52^

Oral steroid intake is also proven to be effective for reducing strictures after esophageal ESD.^53^, ^54^, ^55^, ^56^ Although the dose and duration varies across the studies, results from a recent network meta‐analysis suggest that long‐term (≥12 week) oral steroid intake appears to be an optimal method to prevent strictures after esophageal ESD among steroid application.^57^ Iizuka et al.^58^ also reported that 18‐week oral steroid intake (30 mg prednisolone for 3 weeks and reduction in 5 mg decrements every 3 weeks) showed a significantly lower rate of strictures than 8‐week oral steroid intake (30 mg prednisolone for 2 weeks and tapering) after entire circumferential ESD (36% vs. 82%), although local steroid injection was added as needed in both treatment groups. However, it has been noted that long‐term or higher dose systemic steroid administration can cause several side effects including infection and diabetes mellitus.^59^, ^60^ In fact, a case report indicated a risk of life‐threatening infection when taking oral steroid after esophageal ESD.^61^

## GASTRIC ESD

Gastric ESD has replaced EMR as a standard method for endoscopic resection for early‐stage gastric tumors in Eastern Asian countries. Indeed, over 90% of endoscopic resections for early gastric cancers are ESD in Japan.^62^ In gastric ESD, the major complications include intraoperative and postoperative perforation, delayed bleeding, thromboembolism, and stricture (Table 2).

**TABLE 2:** Major complications and management for preventing them in gastric endoscopic submucosal dissection (ESD)

	Frequency	Preventative method
Intraoperative perforation	2.3% (3.2% of such cases required emergency surgery)^64^	Traction‐assisted method^73^
Delayed perforation	0.4% (35.0% of such cases required emergency surgery)^64^	Prevention of excessive thermal damage on muscularis propria^75^
Delayed bleeding	4.1%–8.5%^66^, ^76^, ^77^, ^78^, ^79^, ^80^	The use of vonoprazan ≥20 mg/day^34^
Thromboembolism	0.03%^91^	Continuation of antiplatelet agents?^80^, ^91^, ^92^, ^93^, ^94^
Stricture	21.3% in cardiac resection 3.2% in antral resection^100^	No established method

### Perforation

A meta‐analysis that included 24,855 patients reported a rate of intraoperative perforation in gastric ESD as 2.7% (95% CI, 2.1%–3.3%).^63^ According to a large‐scale multicenter prospective study in Japan,^64^ intraoperative perforation occurred in 2.3% (218/10,821), but only 3.2% of such cases (7/218) required emergency surgery. Thus, conservative management without surgical intervention is sufficient in most cases with intraoperative perforation. Many risk factors for intraoperative perforation, including invasion depth and submucosal fibrosis, have been reported. Among them, the upper‐third of the stomach and longer procedure time might be especially important since many reports have identified them as risk factors.^65^, ^66^, ^67^, ^68^, ^69^, ^70^, ^71^, ^72^ Obviously, endoscopist‐related factors as well as tumor‐related factors would affect the incidence of perforation. Also, in gastric ESD, traction‐assisted ESD is useful for preventing intraoperative perforation. A multicenter randomized controlled trial demonstrated a lower incidence of intraoperative perforation in traction‐assisted ESD using dental floss than that in conventional ESD (2.2% vs. 0.3%).^73^

Delayed perforation is generally considered as a more serious complication than intraoperative perforation. In fact, although delayed perforation occurred in 0.4% of cases with gastric ESD, 35.0% of such cases required emergency surgery.^64^ The discrepancy in the rate of emergency surgery between intraoperative and delayed perforation may be due to the larger sized perforation and the condition after starting a meal in delayed perforation. To date, the small number of such complications has made it difficult to investigate risk factors; however, a previous report identified that gastric tube cases were significantly associated with delayed perforation.^74^ Furthermore, a possible cause of delayed perforation is necrosis of the muscularis layer due to excessive thermal damage of this layer.^75^ Therefore, to prevent delayed perforation, excessive coagulation should be avoided. If the muscularis layer is excessively coagulated, closure of the ESD ulcer might be useful for preventing delayed perforation.

### Delayed bleeding

**FIGURE 2:**
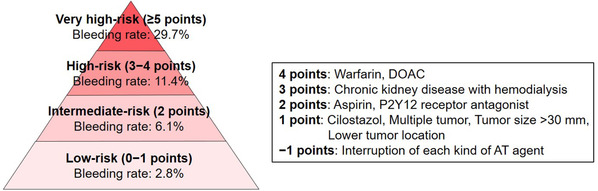
BEST‐J score for predicting delayed bleeding risk after endoscopic submucosal dissection (ESD) for early gastric cancer. AT, antithrombotic; DOAC, direct oral anticoagulant

Delayed bleeding is the most common complication in gastric ESD and this adverse event is reported to occur in 4.1%–8.5% of cases.^66^, ^76^, ^77^, ^78^, ^79^, ^80^ However, the risk of delayed bleeding differs depending on various factors, such as anticoagulants.^81^ Recently, a novel prediction model (BEST‐J score) for delayed bleeding in ESD for early gastric cancers was established.^82^ In this model, points were assigned to factors: 4 points each for warfarin and direct oral anticoagulants (DOACs), 3 points for chronic kidney disease with hemodialysis, 2 points each for P2Y12 receptor antagonist and aspirin, 1 point each for cilostazol, a tumor size >30 mm, lower‐third in tumor location, and the presence of multiple tumors, and −1 point for the interruption of each kind of antithrombotic (AT) agents (Figure 2). The rates of bleeding for low‐ (0–1 points), intermediate‐ (2 points), high‐ (3–4 points), and very high‐risk (≥5 points) categories were 2.8%, 6.1%, 11.4%, and 29.7%, respectively (Figure 2). A mobile application of this model was also developed (https://apps.apple.com/app/id1492914336 for iOS, https://play.google.com/store/apps/details?id=hatta.best_j for Android). Based on the results in this study, anticoagulants have the highest risk for delayed bleeding with a similar risk between warfarin and DOACs, followed by hemodialysis and antiplatelet agents, in gastric ESD.

Then, how can clinicians prevent delayed bleeding especially in patients at high risk? Several methods, such as the use of polyglycolic acid (PGA) sheet,^83^ closure with clips,^84^ and second‐look endoscopy (SLE),^85^ have been proposed. However, most methods had no significant effect in reducing delayed bleeding in gastric ESD (Table 3).^82^, ^86^, ^87^ Regarding SLE, noninferiority of patients with non‐SLE compared to those with SLE was also confirmed in a randomized trial.^88^ A recent database analysis using propensity‐score methods demonstrated that vonoprazan had a significant reducing effect of approximately 30% on delayed bleeding compared with PPI (Table 3).^34^ Furthermore, vonoprazan ≥20 mg/day, but not <20 mg/day, showed a reduced risk of bleeding in comparison with standard/high‐dose PPI in gastroduodenal ESD. Similar results were achieved when gastroduodenal ESD was limited to ESD for gastric tumors. Therefore, vonoprazan ≥20 mg/day would be useful for reducing delayed bleeding in gastric ESD. However, it should be noted that this study has some limitations due to the nature of a retrospective database analysis, such as unmeasured confounder and potential inaccuracy of coding.

**TABLE 3:** Reports about preventative method for delayed bleeding in gastric endoscopic submucosal dissection (ESD)

Author, year	Study population	Preventative method	Control	Study design	No. of cases	Results for delayed bleeding (bleeding rate, preventative method vs. control)
Kataoka et al., 2019^86^	Patients on AT agents or those with large mucosal resection	PGA sheet	Non‐PGA sheet	RCT	137	No significant difference (4.5% vs. 5.7%)
Ego et al., 2020^87^	Patients on AT agents	Mucosal closure	Nonclosure	Retrospective cohort study	400	No significant difference (11.5% vs. 11.9%)
Hatta et al., 2021^82^	All patients	SLE	Non‐SLE	Retrospective case–control study	10,319	No significant difference 2.7% vs. 3.1% in low risk 5.7% vs. 5.9% in intermediate risk 12.9% vs. 9.6% in high risk 29.0% vs. 33.3% in very high risk
Abe et al., 2021^34^	Patients on vonoprazan or PPI	Vonoprazan	PPI	Retrospective cohort study using database (PS matching)	39,740	Reducing effect in the use of vonoprazan (5.4% vs. 7.5%)

Abbreviations: AT, antithrombotic; PGA, polyglycolic acid; PPI, proton pump inhibitor; PS, propensity score; RCT, randomized controlled trial; SLE, second‐look endoscopy.

**FIGURE 3:**
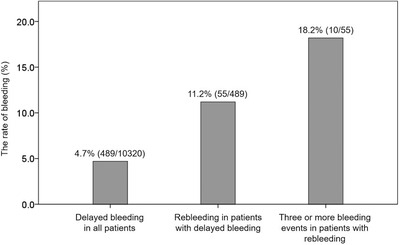
Increased bleeding risk in the cases with repeated bleeding after endoscopic submucosal dissection (ESD) for early gastric cancer. The rate of repeated bleeding gradually increases as the number of bleeding events experienced increases

In patients with delayed bleeding, clinicians should be careful for further bleeding. According to the largest study to date,^89^, ^90^ the rate of delayed bleeding rate was 4.7% (489/10,320), and rebleeding occurred in 11.2% (55/489) of patients with delayed bleeding (Figure 3). Furthermore, 18.2% (10/55) of patients with rebleeding underwent further bleeding (Figure 3). Thus, the risk of repeated bleeding might gradually increase as the number of bleeding events increases.

### Thromboembolism

Regarding thromboembolism in the perioperative period of gastric ESD, only several cases have been reported (Table 4).^80^, ^90^, ^91^, ^92^, ^93^, ^94^ According to a recent large‐scale study by Shiroma et al.,^90^ the rate of thromboembolism in gastric ESD was 0.03% (3/10,320). In reports published until 2017,^80^, ^91^, ^92^, ^93^, ^94^ most patients who underwent thromboembolism took antiplatelet agents with their discontinuation at the time of the thromboembolic events. These studies included a lot of patients who were treated under the discontinuation of antiplatelet agents because the Japanese guidelines recommended discontinuation of them until 2012. By contrast in a study by Shiroma et al. (study period, 2013–2016),^90^ the rate of patients who discontinued antiplatelet agents in the perioperative period of gastric ESD was rather low (6.3%) owing to the change of recommendation for their management in the guidelines,^95^ and no thromboembolic events occurred in those with antiplatelet agents (0/1428). Thus, the change of the management of patients with antiplatelet agents (i.e., no discontinuation of these agents) might have reduced the frequency of this complication in gastric ESD. Meanwhile, anticoagulants were prescribed in all three patients with thromboembolism in the recent study.^90^ In 2017, the management of patients with anticoagulants in the guidelines was further changed^96^ because of the continuing risk of major bleeding in heparin replacement of anticoagulants without any significant effect for preventing thromboembolism.^97^ However, the knowledge of delayed bleeding and thromboembolism in patients with anticoagulants after the latest guidelines is still not sufficient; thus, further studies in this field are required.

**TABLE 4:** Reports about thromboembolism in gastric endoscopic submucosal dissection (ESD)

Author, year	Study population	No. of cases	No. of events (rate)	Type of thromboembolic event	Status of AT agents in cases with event
Lim et al., 2012^80^	All patients	1503	1 (0.07%)	Cerebral infarction	Discontinuation of antiplatelet agents
Takeuchi et al., 2013^94^	All patients	833	1 (0.12%)	Cerebral infarction	Discontinuation of AT agent (detail unclear)
Yoshio et al., 2013^93^	All patients	1250	1 (0.08%)	Cerebral infarction	Discontinuation of aspirin, P2Y12 receptor antagonist, and cilostazol with heparin bridging
Sanomura et al., 2014^92^	Patients on aspirin	78	4 (5.1%)	2, cerebral infarction; 2, myocardial infarction	All, discontinuation of aspirin
Igarashi et al., 2017^91^	Patients on AT agents	367	4 (1.1%)	2, cerebral infarction; 1, TIA; 1, angina pectoris	All, discontinuation of antiplatelet agents
Shiroma et al., 2021^90^	All patients	10,320	3 (0.03%)	2, cerebral infarction; 1, TIA	1, discontinuation of warfarin; 1, discontinuation of warfarin with heparin bridging; 1, continuation of DOAC with heparin bridging

Abbreviations: AT, antithrombotic; DOAC, direct oral anticoagulant; TIA, transient ischemic attack.

### Stricture

The most frequent location of stricture after gastric ESD is the cardia, followed by the antrum.^98^, ^99^ Over three‐fourths of the circumferential extent^99^, ^100^ and extension of the mucosal defect to pyloric ring^100^, ^101^ were independent risk factors for gastric stricture.

Although EBD and steroid application are well‐established preventative methods for stricture in esophageal ESD, their effect in gastric ESD remains unclear. Kishida et al.^100^ reported that early steroid treatment did not have a significant effect on stricture prevention after wide gastric ESD. Sumiyoshi et al.^99^ compared the clinical characteristics between cases with and without prophylactic EBD. However, all were single‐institution studies with small numbers of cases; thus, it is difficult to reach a definite conclusion. A multicenter study with a larger cohort is required for confirming the effect of preventative methods. Furthermore, surgical resection might be more appropriate as a therapeutic approach when suspecting high risk for refractory strictures after ESD, such as an entire circumferential lesion of the pyloric ring.

## DUODENAL ESD

Duodenal ESD is technically more difficult and has greater risk of complications than ESD in other organs. Several reasons for the difficulty in this procedure have been raised.^102^, ^103^ First, since the duodenal wall is thinner, the muscular layer is more vulnerable to electric damage. Second, the presence of Brunner's glands leads to poor submucosal elevation after submucosal injection. Third, the curved shape of the duodenum and its narrow lumen reduce the maneuverability of the endoscope. In addition, exposure to pancreatic and bile juice is also problematic. Thus, this procedure should be confined to endoscopists with extensive experience in performing ESD in other organs.^104^ The major complications in duodenal ESD are intraoperative and delayed perforation and delayed bleeding (Table 5). With the development of devices and techniques, the frequency of complications in duodenal ESD has been decreasing.

**TABLE 5:** Major complications and management for preventing them in duodenal endoscopic submucosal dissection (ESD)

	Frequency	Preventative method
Intraoperative perforation	6.0%–31.6% (emergency surgery was required in 3.1%–23.1% of such cases)^105^, ^106^, ^107^, ^108^, ^109^	The use of the Clutch Cutter^105^ Pocket‐creation method^106^ Water pressure method^111^
Delayed perforation	1.5%–4.8%^105^, ^106^, ^107^, ^108^, ^109^ (emergency surgery was required in 25.0%–100.0% of such cases^106^, ^107^, ^108^, ^109^)	Mucosal defect closure (clips, clips with string, an endoloop, over‐the‐scope clips)^105^, ^112^, ^113^, ^114^ Coverage of mucosal defect with a PGA sheet^115^, ^116^
Delayed bleeding	0.0%–18.4%^105^, ^106^, ^108^, ^109^, ^118^	Mucosal defect closure (clips, clips with string, an endoloop, over‐the‐scope clips)^117^ Coverage of mucosal defect with PGA sheet^117^ The use of vonoprazan ≥20 mg/day^34^

Abbreviation: PGA, polyglycolic acid.

### Perforation

The rate of intraoperative perforation in duodenal ESD was reported to be 6.0%–31.6%,^105^, ^106^, ^107^, ^108^, ^109^ which is about 3–14 times higher than that in gastric ESD.^64^ Emergency surgery was required in 23.1% (6/26) of such cases when combining the results of four studies.^104^, ^105^, ^107^, ^108^ On the other hand, the largest study to date revealed that additional intervention is required in 3.1% of cases with perforation.^107^ This is surprising because the rate of requiring additional intervention in duodenal ESD was similar to those in esophageal and gastric ESD,^64^, ^110^ although it should be noted that differences in the study period may have affected the frequency of perforation and additional intervention in duodenal ESD. Furthermore, when complete mucosal closure was achieved, no cases required additional intervention and the clinical course did not significantly differ between these cases and those without perforation.^107^ Meanwhile, in patients in whom the lesions are located distal to the superior duodenal angle and complete mucosal closure cannot be achieved after perforation, an endoscopic nasobiliary and pancreatic duct drainage tube, and preventing the exposure of pancreatic and bile juice to the mucosal defect may be effective in preventing a worse clinical course.^107^ To reduce intraoperative perforation, several methods have been proposed. Dohi et al.^105^ reported that no perforation occurred in 47 cases with duodenal ESD using the Clutch Cutter, which is one of the scissors‐type knives. Using this knife in ESD is relatively simple and safe for dissecting a narrow space and, even if the lesion has severe fibrosis, ESD can be safely performed using the distal tips of the Clutch Cutter.^105^ A pocket‐creation method^106^ and water pressure method^111^ were also developed for safe ESD.

Delayed perforation was reported to occur in 1.5%–4.8% of cases with duodenal ESD,^105^, ^106^, ^107^, ^108^, ^109^ which is about 5–16 times higher than that in gastric ESD.^64^ Emergency surgery was required in all of such cases (3/3) in the results of three studies (no data were available in one study),^106^, ^108^, ^109^ whereas the largest study reported a rate of 25.0% (1/4).^107^ To prevent this complication, various endoscopic mucosal defect closure techniques, including closure with clips, clips with string,^112^ an endoloop,^113^ over‐the‐scope clips,^105^, ^114^ and coverage with a PGA sheet,^115^, ^116^ have been performed. A meta‐analysis revealed that the rates of delayed perforation in patients with and without mucosal defect closure after duodenal endoscopic resection were 1.6% and 3.8%, respectively.^117^ The reduction by mucosal defect closure techniques for perforation did not reach statistical significance (*p* = 0.13) in this meta‐analysis possibly due to the small number of cases, but the risk ratio was low (0.39). Mucosal defect closure techniques have a potential to prevent delayed perforation, and a future large‐scale study or meta‐analysis may demonstrate their significant effect.

### Delayed bleeding

The rate of delayed bleeding is reported to be 0.0%–18.4%.^105^, ^106^, ^108^, ^109^, ^118^ A meta‐analysis revealed that endoscopic mucosal defect closure techniques significantly reduced delayed bleeding in duodenal endoscopic resection (risk ratio, 0.14; delayed bleeding rate, 2.0% vs. 17.3%).^118^ Furthermore, a recent database analysis found that the use of vonoprazan showed a significant reduction of delayed bleeding compared to PPI in ESD for duodenal tumors,^34^ although this study did not consider the effect of endoscopic preventive procedures after ESD. Thus, endoscopic mucosal defect closure techniques should now be mandatory for preventing delayed bleeding, and vonoprazan intake has the possibility to reduce this complication as compared to PPI.

## CONCLUSIONS

We showed the current status of major complications in upper GI ESD and discussed recent approach for preventing them. Although many methods have been developed to prevent complications in esophageal and gastric ESD, several issues, including the prevention of delayed bleeding after gastric ESD, have remained unresolved. For duodenal ESD, which is still challenging in the current status, a future large‐scale study is demanded to accumulate more evidence.

## CONFLICT OF INTEREST

The author W.H. is an associate editor of DEN Open.

## FUNDING INFORMATION

None.

## References

[1] Kim SG , Park CM , Lee NR , *et al*. Long‐term clinical outcomes of endoscopic submucosal dissection in patients with early gastric cancer: A prospective multicenter cohort study. Gut Liver. 2018; 12: 402–10. 29588436 10.5009/gnl17414 PMC6027839

[2] Hatta W , Gotoda T , Koike T , Masamune A . History and future perspectives in Japanese guidelines for endoscopic resection of early gastric cancer. Dig. Endosc. 2020; 32: 180–90. 31529716 10.1111/den.13531

[3] Tsujii Y , Nishida T , Nishiyama O , *et al*. Clinical outcomes of endoscopic submucosal dissection for superficial esophageal neoplasms: A multicenter retrospective cohort study. Endoscopy 2015; 47: 775–83. 25826277 10.1055/s-0034-1391844

[4] Hatta W , Gotoda T , Koike T , Masamune A . A recent argument for the use of endoscopic submucosal dissection for early gastric cancers. Gut Liver. 2020; 14: 412–22. 31554392 10.5009/gnl19194 PMC7366137

[5] Park JS , Youn YH , Park JJ , Kim JH , Park H . Clinical outcomes of endoscopic submucosal dissection for superficial esophageal squamous neoplasms. Clin. Endosc. 2016; 49: 168–75. 26867548 10.5946/ce.2015.080 PMC4821515

[6] Tanabe S , Ishido K , Matsumoto T , *et al*. Long‐term outcomes of endoscopic submucosal dissection for early gastric cancer: A multicenter collaborative study. Gastric Cancer. 2017; 20: 45–52. 27807641 10.1007/s10120-016-0664-7

[7] Hatta W , Gotoda T , Oyama T , *et al*. Is radical surgery necessary in all patients who do not meet the curative criteria for endoscopic submucosal dissection in early gastric cancer? A multi‐center retrospective study in Japan. J. Gastroenterol. 2017; 52: 175–84. 27098174 10.1007/s00535-016-1210-4

[8] Isomoto H , Shikuwa S , Yamaguchi N , *et al*. Endoscopic submucosal dissection for early gastric cancer: A large‐scale feasibility study. Gut 2009; 58: 331–6. 19001058 10.1136/gut.2008.165381

[9] Ogata Y , Hatta W , Koike T , *et al*. Predictors of early and late mortality after endoscopic resection for esophageal squamous cell carcinoma. Tohoku J. Exp. Med. 2021; 253: 29–39. 33441512 10.1620/tjem.253.29

[10] Hatta W , Koike T , Takahashi S , *et al*. Risk of metastatic recurrence after endoscopic resection for esophageal squamous cell carcinoma invading into the muscularis mucosa or submucosa: A multicenter retrospective study. J. Gastroenterol. 2021; 56: 620–32. 33881632 10.1007/s00535-021-01787-y

[11] Dohi O , Hatta W , Gotoda T , *et al*. Long‐term outcomes after non‐curative endoscopic submucosal dissection for early gastric cancer according to hospital volumes in Japan: A multicenter propensity‐matched analysis. Surg. Endosc. 2019; 33: 4078–88. 30805782 10.1007/s00464-019-06710-4

[12] Eguchi T , Nakanishi Y , Shimoda T , *et al*. Histopathological criteria for additional treatment after endoscopic mucosal resection for esophageal cancer: Analysis of 464 surgically resected cases. Mod. Pathol. 2006; 19: 475–80. 16444191 10.1038/modpathol.3800557

[13] Hatta W , Gotoda T , Oyama T , *et al*. A scoring system to stratify curability after endoscopic submucosal dissection for early gastric cancer: “eCura system”. Am. J. Gastroenterol. 2017; 112: 874–81. 28397873 10.1038/ajg.2017.95

[14] Kakushima N , Kanemoto H , Tanaka M , Takizawa K , Ono H . Treatment for superficial non‐ampullary duodenal epithelial tumors. World J. Gastroenterol. 2014; 20: 12501–8. 25253950 10.3748/wjg.v20.i35.12501 PMC4168083

[15] Hatta W , Gotoda T , Kanno T , *et al*. Prevalence and risk factors for lymph node metastasis after noncurative endoscopic resection for early gastric cancer: A systematic review and meta‐analysis. J. Gastroenterol. 2020; 55: 742–53. 32277297 10.1007/s00535-020-01685-9

[16] Yamada S , Hatta W , Shimosegawa T , *et al*. Different risk factors between early and late cancer recurrences in patients without additional surgery after noncurative endoscopic submucosal dissection for early gastric cancer. Gastrointest. Endosc. 2019; 89: 950–60. 30465769 10.1016/j.gie.2018.11.015

[17] Han C , Sun Y . Efficacy and safety of endoscopic submucosal dissection versus endoscopic mucosal resection for superficial esophageal carcinoma: A systematic review and meta‐analysis. Dis. Esophagus. 2021; 34: doaa081. 32895709 10.1093/dote/doaa081

[18] Odagiri H , Yasunaga H , Matsui H , Matsui S , Fushimi K , Kaise M . Hospital volume and adverse events following esophageal endoscopic submucosal dissection in Japan. Endoscopy 2017; 49: 321–6. 27975337 10.1055/s-0042-122189

[19] Noguchi M , Yano T , Kato T , *et al*. Risk factors for intraoperative perforation during endoscopic submucosal dissection of superficial esophageal squamous cell carcinoma. World J. Gastroenterol. 2017; 23: 478–85. 28210084 10.3748/wjg.v23.i3.478 PMC5291853

[20] Oyama T . Esophageal ESD: Technique and prevention of complications. Gastrointest. Endosc. Clin. N. Am. 2014; 24: 201–12. 24679232 10.1016/j.giec.2013.12.001

[21] Yoshida M , Takizawa K , Nonaka S , *et al*. Conventional versus traction‐assisted endoscopic submucosal dissection for large esophageal cancers: A multicenter, randomized controlled trial (with video). Gastrointest. Endosc. 2020; 91: 55–65.e2. 31445039 10.1016/j.gie.2019.08.014

[22] Ishihara R , Arima M , Iizuka T , *et al*. Endoscopic submucosal dissection/endoscopic mucosal resection guidelines for esophageal cancer. Dig. Endosc. 2020; 32: 452–93. 32072683 10.1111/den.13654

[23] Omae M , Konradsson M , Baldaque‐Silva F . Delayed perforation after endoscopic submucosal dissection treated successfully by temporary stent placement. Clin. J. Gastroenterol. 2018; 11: 118–22. 29222735 10.1007/s12328-017-0808-2

[24] Hanaoka N , Uedo N , Ishihara R , *et al*. Clinical features and outcomes of delayed perforation after endoscopic submucosal dissection for early gastric cancer. Endoscopy 2010; 42: 1112–5. 21120780 10.1055/s-0030-1255932

[25] Hirasawa K , Sato C , Makazu M , *et al*. Coagulation syndrome: Delayed perforation after colorectal endoscopic treatments. World J. Gastrointest. Endosc. 2015; 7: 1055–61. 26380051 10.4253/wjge.v7.i12.1055 PMC4564832

[26] Matsuda Y , Kataoka N , Yamaguchi T , Tomita M , Sakamoto K , Makimoto S . Delayed esophageal perforation occurring with endoscopic submucosal dissection: A report of two cases. World J. Gastrointest. Surg. 2015; 7: 123–7. 26225195 10.4240/wjgs.v7.i7.123 PMC4513435

[27] Ono S , Fujishiro M , Niimi K , *et al*. Long‐term outcomes of endoscopic submucosal dissection for superficial esophageal squamous cell neoplasms. Gastrointest. Endosc. 2009; 70: 860–6. 19577748 10.1016/j.gie.2009.04.044

[28] Hirasawa K , Kokawa A , Oka H , *et al*. Superficial adenocarcinoma of the esophagogastric junction: Long‐term results of endoscopic submucosal dissection. Gastrointest. Endosc. 2010; 72: 960–6. 21034897 10.1016/j.gie.2010.07.030

[29] Kawahara Y , Hori K , Takenaka R , *et al*. Endoscopic submucosal dissection of esophageal cancer using the Mucosectom2 device: A feasibility study. Endoscopy 2013; 45: 869–75. 23884795 10.1055/s-0033-1344229

[30] Isomoto H , Yamaguchi N , Minami H , Nakao K . Management of complications associated with endoscopic submucosal dissection/endoscopic mucosal resection for esophageal cancer. Dig. Endosc. 2013; 25: 29–38. 23368404 10.1111/j.1443-1661.2012.01388.x

[31] Kagemoto K , Oka S , Tanaka S , *et al*. Clinical outcomes of endoscopic submucosal dissection for superficial Barrett's adenocarcinoma. Gastrointest. Endosc. 2014; 80: 239–45. 24565073 10.1016/j.gie.2014.01.022

[32] Yang D , Coman RM , Kahaleh M , *et al*. Endoscopic submucosal dissection for Barrett's early neoplasia: A multicenter study in the United States. Gastrointest. Endosc. 2017; 86: 600–7. 27688205 10.1016/j.gie.2016.09.023

[33] Iizuka T , Kikuchi D , Hoteya S . Outcomes of endoscopic submucosal dissection for superficial esophageal cancer in an elderly population: A retrospective single center cohort study. Endosc. Int. Open. 2019; 7: E355–60. 30834294 10.1055/a-0832-8257 PMC6395099

[34] Abe H , Hatta W , Ogata Y , *et al*. Prevention of delayed bleeding with vonoprazan in upper gastrointestinal endoscopic treatment. J. Gastroenterol. 2021; 56: 640–50. 33876324 10.1007/s00535-021-01781-4

[35] Sakurai Y , Mori Y , Okamoto H , *et al*. Acid‐inhibitory effects of vonoprazan 20 mg compared with esomeprazole 20 mg or rabeprazole 10 mg in healthy adult male subjects—a randomised open‐label cross‐over study. Aliment. Pharmacol. Ther. 2015; 42: 719–30. 26193978 10.1111/apt.13325

[36] Takahashi K , Sato Y , Takeuchi M , *et al*. Changes in esophageal motility after endoscopic submucosal dissection for superficial esophageal cancer: A high‐resolution manometry study. Dis. Esophagus. 2017; 30: 1–8. 10.1093/dote/dox057 28881900

[37] Brunner G , Luna P , Thiesemann C . Drugs for pH control in upper gastrointestinal bleeding. Aliment. Pharmacol. Ther. 1995; 9: 47–50. 10.1111/j.1365-2036.1995.tb00784.x 7495943

[38] Hatta W , Koike T , Okata H , *et al*. Continuous liquid‐suction catheter attachment for endoscope reduces volume of liquid reflux to the mouth in esophageal endoscopic submucosal dissection. Dig. Endosc. 2019; 31: 527–34. 30861606 10.1111/den.13392

[39] Muramoto T , Aoki A , Suzuki Y , Hishida M , Ohata K . Continuous saliva suction tube to prevent aspiration pneumonia during upper GI endoscopy. VideoGIE. 2021; 6: 114–5. 33738357 10.1016/j.vgie.2020.11.002 PMC7947250

[40] Maekita T , Kato J , Nakatani Y , *et al*. Usefulness of a continuous suction mouthpiece during percutaneous endoscopic gastrostomy: A single‐center, prospective, randomized study. Dig. Endosc. 2013; 25: 496–501. 23368904 10.1111/den.12017

[41] Hatta W , Gotoda T , Koike T , Masamune A . Management following endoscopic resection in elderly patients with early‐stage upper gastrointestinal neoplasia. Dig. Endosc. 2020; 32: 861–73. 31802529 10.1111/den.13592

[42] Ono S , Fujishiro M , Niimi K , *et al*. Predictors of postoperative stricture after esophageal endoscopic submucosal dissection for superficial squamous cell neoplasms. Endoscopy 2009; 41: 661–5. 19565442 10.1055/s-0029-1214867

[43] Shi Q , Ju H , Yao LQ , *et al*. Risk factors for postoperative stricture after endoscopic submucosal dissection for superficial esophageal carcinoma. Endoscopy 2014; 46: 640–4. 24830402 10.1055/s-0034-1365648

[44] Ezoe Y , Muto M , Horimatsu T , *et al*. Efficacy of preventive endoscopic balloon dilation for esophageal stricture after endoscopic resection. J. Clin. Gastroenterol. 2011; 45: 222–7. 20861798 10.1097/MCG.0b013e3181f39f4e

[45] Hashimoto S , Kobayashi M , Takeuchi M , Sato Y , Narisawa R , Aoyagi Y . The efficacy of endoscopic triamcinolone injection for the prevention of esophageal stricture after endoscopic submucosal dissection. Gastrointest. Endosc. 2011; 74: 1389–93. 22136782 10.1016/j.gie.2011.07.070

[46] Hanaoka N , Ishihara R , Takeuchi Y , *et al*. Intralesional steroid injection to prevent stricture after endoscopic submucosal dissection for esophageal cancer: A controlled prospective study. Endoscopy 2012; 44: 1007–11. 22930171 10.1055/s-0032-1310107

[47] Nagami Y , Shiba M , Tominaga K , *et al*. Locoregional steroid injection prevents stricture formation after endoscopic submucosal dissection for esophageal cancer: A propensity score matching analysis. Surg. Endosc. 2016; 30: 1441–9. 26123341 10.1007/s00464-015-4348-x

[48] Takahashi H , Arimura Y , Okahara S , *et al*. A randomized controlled trial of endoscopic steroid injection for prophylaxis of esophageal stenoses after extensive endoscopic submucosal dissection. BMC Gastroenterol. 2015; 15: 1. 25609176 10.1186/s12876-014-0226-6 PMC4308850

[49] Kadota T , Yano T , Kato T , *et al*. Prophylactic steroid administration for strictures after endoscopic resection of large superficial esophageal squamous cell carcinoma. Endosc. Int. Open. 2016; 4: E1267–74. 28028531 10.1055/s-0042-118291 PMC5179327

[50] Nagami Y , Shiba M , Ominami M , *et al*. Single locoregional triamcinolone injection immediately after esophageal endoscopic submucosal dissection prevents stricture formation. Clin. Transl. Gastroenterol. 2017; 8: e75. 28230852 10.1038/ctg.2017.5 PMC5387750

[51] Tsujii Y , Hayashi Y , Kawai N , *et al*. Risk of perforation in balloon dilation associated with steroid injection for preventing esophageal stricture after endoscopic submucosal dissection. Endosc. Int. Open. 2017; 5: E573–9. 28670613 10.1055/s-0043-110077 PMC5482748

[52] Nagami Y , Ominami M , Shiba M , *et al*. Prediction of esophageal stricture in patients given locoregional triamcinolone injections immediately after endoscopic submucosal dissection. Dig. Endosc. 2018; 30: 198–205. 28803459 10.1111/den.12946

[53] Yamaguchi N , Isomoto H , Nakayama T , *et al*. Usefulness of oral prednisolone in the treatment of esophageal stricture after endoscopic submucosal dissection for superficial esophageal squamous cell carcinoma. Gastrointest. Endosc. 2011; 73: 1115–21. 21492854 10.1016/j.gie.2011.02.005

[54] Sato H , Inoue H , Kobayashi Y , *et al*. Control of severe strictures after circumferential endoscopic submucosal dissection for esophageal carcinoma: Oral steroid therapy with balloon dilation or balloon dilation alone. Gastrointest. Endosc. 2013; 78: 250–7. 23453294 10.1016/j.gie.2013.01.008

[55] Zhou G , Yuan F , Cai J , *et al*. Efficacy of prednisone for prevention of esophageal stricture after endoscopic submucosal dissection for superficial esophageal squamous cell carcinoma. Thorac. Cancer. 2017; 8: 489–94. 28759148 10.1111/1759-7714.12473 PMC5582460

[56] Kataoka M , Anzai S , Shirasaki T , *et al*. Efficacy of short period, low dose oral prednisolone for the prevention of stricture after circumferential endoscopic submucosal dissection (ESD) for esophageal cancer. Endosc. Int. Open. 2015; 3: E113–7. 26135649 10.1055/s-0034-1390797 PMC4477014

[57] Yang J , Wang X , Li Y , *et al*. Efficacy and safety of steroid in the prevention of esophageal stricture after endoscopic submucosal dissection: A network meta‐analysis. J. Gastroenterol. Hepatol. 2019; 34: 985–95. 30566746 10.1111/jgh.14580

[58] Iizuka T , Kikuchi D , Hoteya S , Kaise M . Effectiveness of modified oral steroid administration for preventing esophageal stricture after entire circumferential endoscopic submucosal dissection. Dis. Esophagus. 2018; 31. 10.1093/dote/dox140 29444278

[59] Klein NC , Go CH , Cunha BA . Infections associated with steroid use. Infect. Dis. Clin. N. Am. 2001; 15: 423–32, viii. 10.1016/s0891-5520(05)70154-9 11447704

[60] Hoes JN , Jacobs JWG , Boers M , *et al*. EULAR evidence‐based recommendations on the management of systemic glucocorticoid therapy in rheumatic diseases. Ann. Rheum. Dis. 2007; 66: 1560–7. 17660219 10.1136/ard.2007.072157 PMC2095301

[61] Ishida T , Morita Y , Hoshi N, *et al*. Disseminated nocardiosis during systemic steroid therapy for the prevention of esophageal stricture after endoscopic submucosal dissection. Dig. Endosc. 2015; 27: 388–91. 24889691 10.1111/den.12317

[62] Fujishiro M , Yoshida S , Matsuda R , Narita A , Yamashita H , Seto Y . Updated evidence on endoscopic resection of early gastric cancer from Japan. Gastric Cancer. 2017; 20: 39–44. 27704225 10.1007/s10120-016-0647-8

[63] Akintoye E , Obaitan I , Muthusamy A , Akanbi O , Olusunmade M , Levine D . Endoscopic submucosal dissection of gastric tumors: A systematic review and meta‐analysis. World J. Gastrointest. Endosc. 2016; 8: 517–32. 27606044 10.4253/wjge.v8.i15.517 PMC4980641

[64] Suzuki H , Takizawa K , Hirasawa T , *et al*. Short‐term outcomes of multicenter prospective cohort study of gastric endoscopic resection: ‘Real‐world evidence’ in Japan. Dig. Endosc. 2019; 31: 30–9. 30058258 10.1111/den.13246

[65] Imagawa A , Okada H , Kawahara Y , *et al*. Endoscopic submucosal dissection for early gastric cancer: Results and degrees of technical difficulty as well as success. Endoscopy 2006; 38: 987–90. 17058162 10.1055/s-2006-944716

[66] Toyokawa T , Inaba T , Omote S , *et al*. Risk factors for perforation and delayed bleeding associated with endoscopic submucosal dissection for early gastric neoplasms: Analysis of 1123 lesions. J. Gastroenterol. Hepatol. 2012; 27: 907–12. 22142449 10.1111/j.1440-1746.2011.07039.x

[67] Yamaguchi N , Isomoto H , Fukuda E , *et al*. Clinical outcomes of endoscopic submucosal dissection for early gastric cancer by indication criteria. Digestion 2009; 80: 173–81. 19776581 10.1159/000215388

[68] Ohta T , Ishihara R , Uedo N , *et al*. Factors predicting perforation during endoscopic submucosal dissection for gastric cancer. Gastrointest. Endosc. 2012; 75: 1159–65. 22482916 10.1016/j.gie.2012.02.015

[69] Kim HJ , Chung H , Jung DH , *et al*. Clinical outcomes of and management strategy for perforations associated with endoscopic submucosal dissection of an upper gastrointestinal epithelial neoplasm. Surg. Endosc. 2016; 30: 5059–67. 26983439 10.1007/s00464-016-4854-5

[70] Mannen K , Tsunada S , Hara M , *et al*. Risk factors for complications of endoscopic submucosal dissection in gastric tumors: Analysis of 478 lesions. J. Gastroenterol. 2010; 45: 30–6. 19760133 10.1007/s00535-009-0137-4

[71] Yoo JH , Shin SJ , Lee KM , *et al*. Risk factors for perforations associated with endoscopic submucosal dissection in gastric lesions: Emphasis on perforation type. Surg. Endosc. 2012; 26: 2456–64. 22398962 10.1007/s00464-012-2211-x

[72] Ojima T , Takifuji K , Nakamura M , *et al*. Complications of endoscopic submucosal dissection for gastric noninvasive neoplasia: An analysis of 647 lesions. Surg. Laparosc. Endosc. Percutan. Tech. 2014; 24: 370–4. 24710239 10.1097/SLE.0b013e318290132e

[73] Yoshida M , Takizawa K , Suzuki S , et al. Conventional versus traction‐assisted endoscopic submucosal dissection for gastric neoplasms: A multicenter, randomized controlled trial (with video). Gastrointest. Endosc. 2018; 87: 1231–40. 29233673 10.1016/j.gie.2017.11.031

[74] Suzuki H , Oda I , Sekiguchi M , *et al*. Management and associated factors of delayed perforation after gastric endoscopic submucosal dissection. World J. Gastroenterol. 2015; 21: 12635–43. 26640340 10.3748/wjg.v21.i44.12635 PMC4658618

[75] Saito I , Tsuji Y , Sakaguchi Y , *et al*. Complications related to gastric endoscopic submucosal dissection and their managements. Clin. Endosc. 2014; 47: 398–403. 25324997 10.5946/ce.2014.47.5.398 PMC4198554

[76] Yano T , Tanabe S , Ishido K , *et al*. Different clinical characteristics associated with acute bleeding and delayed bleeding after endoscopic submucosal dissection in patients with early gastric cancer. Surg. Endosc. 2017; 31: 4542–50. 28378078 10.1007/s00464-017-5513-1

[77] Sato C , Hirasawa K , Koh R , *et al*. Postoperative bleeding in patients on antithrombotic therapy after gastric endoscopic submucosal dissection. World J. Gastroenterol. 2017; 23: 5557–66. 28852315 10.3748/wjg.v23.i30.5557 PMC5558119

[78] Miyahara K , Iwakiri R , Shimoda R , *et al*. Perforation and postoperative bleeding of endoscopic submucosal dissection in gastric tumors: Analysis of 1190 lesions in low‐ and high‐volume centers in Saga, Japan. Digestion. 2012; 86: 273–80. 22986899 10.1159/000341422

[79] Nam HS , Choi CW , Kim SJ , *et al*. Risk factors for delayed bleeding by onset time after endoscopic submucosal dissection for gastric neoplasm. Sci. Rep. 2019; 9: 2674. 30804386 10.1038/s41598-019-39381-1 PMC6389879

[80] Lim JH , Kim SG , Kim JW , *et al*. Do antiplatelets increase the risk of bleeding after endoscopic submucosal dissection of gastric neoplasms? Gastrointest. Endosc. 2012; 75: 719–27. 22317881 10.1016/j.gie.2011.11.034

[81] Tomida H , Yoshio T , Igarashi K , *et al*. Influence of anticoagulants on the risk of delayed bleeding after gastric endoscopic submucosal dissection: A multicenter retrospective study. Gastric Cancer. 2021; 24: 179–89. 32683602 10.1007/s10120-020-01105-0

[82] Hatta W , Tsuji Y , Yoshio T , *et al*. Prediction model of bleeding after endoscopic submucosal dissection for early gastric cancer: BEST‐J score. Gut. 2021; 70: 476–84. 32499390 10.1136/gutjnl-2019-319926 PMC7873424

[83] Tsuji Y , Fujishiro M , Kodashima S , *et al*. Polyglycolic acid sheets and fibrin glue decrease the risk of bleeding after endoscopic submucosal dissection of gastric neoplasms (with video). Gastrointest. Endosc. 2015; 81: 906–12. 25440679 10.1016/j.gie.2014.08.028

[84] Zhang QS , Han B , Xu JH , Gao P , Shen YC . Clip closure of defect after endoscopic resection in patients with larger colorectal tumors decreased the adverse events. Gastrointest. Endosc. 2015; 82: 904–9. 25975527 10.1016/j.gie.2015.04.005

[85] Kim HH , Park SJ , Park MI , Moon W . Clinical impact of second‐look endoscopy after endoscopic submucosal dissection of gastric neoplasms. Gut Liver. 2012; 6: 316–20. 22844558 10.5009/gnl.2012.6.3.316 PMC3404167

[86] Kataoka Y , Tsuji Y , Hirasawa K , *et al*. Endoscopic tissue shielding to prevent bleeding after endoscopic submucosal dissection: A prospective multicenter randomized controlled trial. Endoscopy 2019; 51: 619–27. 30861532 10.1055/a-0860-5280

[87] Ego M , Abe S , Nonaka S , *et al*. Endoscopic closure utilizing endoloop and endoclips after gastric endoscopic submucosal dissection for patients on antithrombotic therapy. Dig. Dis. Sci. 2021; 66: 2336–44. 32797345 10.1007/s10620-020-06508-8

[88] Mochizuki S , Uedo N , Oda I , *et al*. Scheduled second‐look endoscopy is not recommended after endoscopic submucosal dissection for gastric neoplasms (the SAFE trial): A multicentre prospective randomised controlled non‐inferiority trial. Gut 2015; 64: 397–405. 25301853 10.1136/gutjnl-2014-307552

[89] Hashimoto M , Hatta W , Tsuji Y , *et al*. Rebleeding in patients with delayed bleeding after endoscopic submucosal dissection for early gastric cancer. Dig. Endosc. Published online: 4 Feb 2021; DOI: 10.1111/den.13943 33539035

[90] Shiroma S , Hatta W , Tsuji Y , *et al*. Timing of bleeding and thromboembolism associated with endoscopic submucosal dissection for gastric cancer in Japan. J. Gastroenterol. Hepatol. Published online: 7 May 2021; DOI: 10.1111/jgh.15536 33960518

[91] Igarashi K , Takizawa K , Kakushima N , *et al*. Should antithrombotic therapy be stopped in patients undergoing gastric endoscopic submucosal dissection? Surg. Endosc. 2017; 31: 1746–53. 27530896 10.1007/s00464-016-5167-4

[92] Sanomura Y , Oka S , Tanaka S , *et al*. Continued use of low‐dose aspirin does not increase the risk of bleeding during or after endoscopic submucosal dissection for early gastric cancer. Gastric Cancer. 2014; 17: 489–96. 24142107 10.1007/s10120-013-0305-3 PMC4072060

[93] Yoshio T , Nishida T , Kawai N , *et al*. Gastric ESD under heparin replacement at high‐risk patients of thromboembolism is technically feasible but has a high risk of delayed bleeding: Osaka University ESD Study Group. Gastroenterol. Res. Pract. 2013; 2013: 365830. 23843783 10.1155/2013/365830 PMC3697307

[94] Takeuchi T , Ota K , Harada S , *et al*. The postoperative bleeding rate and its risk factors in patients on antithrombotic therapy who undergo gastric endoscopic submucosal dissection. BMC Gastroenterol. 2013; 13: 136. 24010587 10.1186/1471-230X-13-136 PMC3844538

[95] Fujimoto K , Fujishiro M , Kato M , *et al*. Guidelines for gastroenterological endoscopy in patients undergoing antithrombotic treatment. Dig. Endosc. 2014; 26: 1–14. 10.1111/den.12183 24215155

[96] Kato M , Uedo N , Hokimoto S , *et al*. Guidelines for gastroenterological endoscopy in patients undergoing antithrombotic treatment: 2017 appendix on anticoagulants including direct oral anticoagulants. Dig. Endosc. 2018; 30: 433–40. 29733468 10.1111/den.13184

[97] Douketis JD , Spyropoulos AC , Kaatz S , *et al*. Perioperative bridging anticoagulation in patients with atrial fibrillation. N. Engl. J. Med. 2015; 373: 823–33. 26095867 10.1056/NEJMoa1501035 PMC4931686

[98] Coda S , Oda I , Gotoda T , Yokoi C , Kikuchi T , Ono H . Risk factors for cardiac and pyloric stenosis after endoscopic submucosal dissection, and efficacy of endoscopic balloon dilation treatment. Endoscopy 2009; 41: 421–6. 19418396 10.1055/s-0029-1214642

[99] Sumiyoshi T , Kondo H , Minagawa T , *et al*. Risk factors and management for gastric stenosis after endoscopic submucosal dissection for gastric epithelial neoplasm. Gastric Cancer. 2017; 20: 690–8. 27905029 10.1007/s10120-016-0673-6

[100] Kishida Y , Kakushima N , Takizawa K , *et al*. Effects of steroid use for stenosis prevention after wide endoscopic submucosal dissection for gastric neoplasm. Surg. Endosc. 2018; 32: 751–9. 28733736 10.1007/s00464-017-5732-5

[101] Lee JU , Park MS , Yun SH , *et al*. Risk factors and management for pyloric stenosis occurred after endoscopic submucosal dissection adjacent to pylorus. Medicine (Baltimore). 2016; 95: e5633. 27977608 10.1097/MD.0000000000005633 PMC5268054

[102] Akahoshi K , Kubokawa M , Inamura K , Akahoshi K , Shiratsuchi Y , Tamura S . Current challenge: Endoscopic submucosal dissection of superficial non‐ampullary duodenal epithelial tumors. Curr. Treat. Options Oncol. 2020; 21: 98. 33104938 10.1007/s11864-020-00796-y PMC7588384

[103] Libanio D , Pimentel‐Nunes P , Dinis‐Ribeiro M . Complications of endoscopic resection techniques for upper GI tract lesions. Best Pract. Res. Clin. Gastroenterol. 2016; 30: 735–48. 27931633 10.1016/j.bpg.2016.09.010

[104] Draganov PV , Wang AY , Othman MO , Fukami N . AGA institute clinical practice update: Endoscopic submucosal dissection in the United States. Clin. Gastroenterol. Hepatol. 2019; 17: 16–25.e1. 30077787 10.1016/j.cgh.2018.07.041

[105] Dohi O , Yoshida N , Naito Y , *et al*. Efficacy and safety of endoscopic submucosal dissection using a scissors‐type knife with prophylactic over‐the‐scope clip closure for superficial non‐ampullary duodenal epithelial tumors. Dig. Endosc. 2020; 32: 904–13. 31883154 10.1111/den.13618

[106] Miura Y , Shinozaki S , Hayashi Y , Sakamoto H , Lefor AK , Yamamoto H . Duodenal endoscopic submucosal dissection is feasible using the pocket‐creation method. Endoscopy 2017; 49: 8–14. 27875854 10.1055/s-0042-116315

[107] Fukuhara S , Kato M , Iwasaki E , *et al*. Management of perforation related to endoscopic submucosal dissection for superficial duodenal epithelial tumors. Gastrointest. Endosc. 2020; 91: 1129–37. 31563595 10.1016/j.gie.2019.09.024

[108] Hoteya S , Yahagi N , Iizuka T , *et al*. Endoscopic submucosal dissection for nonampullary large superficial adenocarcinoma/adenoma of the duodenum: Feasibility and long‐term outcomes. Endosc. Int. Open. 2013; 1: 2–7. 26135505 10.1055/s-0033-1359232 PMC4440373

[109] Yamamoto Y , Yoshizawa N , Tomida H , Fujisaki J , Igarashi M . Therapeutic outcomes of endoscopic resection for superficial non‐ampullary duodenal tumor. Dig. Endosc. 2014; 26: 50–6. 24750149 10.1111/den.12273

[110] Daoud DC , Suter N , Durand M , Bouin M , Faulques B , von Renteln D . Comparing outcomes for endoscopic submucosal dissection between Eastern and Western countries: A systematic review and meta‐analysis. World J. Gastroenterol. 2018; 24: 2518–36. 29930473 10.3748/wjg.v24.i23.2518 PMC6010943

[111] Yahagi N , Nishizawa T , Sasaki M , Ochiai Y , Uraoka T . Water pressure method for duodenal endoscopic submucosal dissection. Endoscopy 2017; 49: E227–8. 28759932 10.1055/s-0043-113556

[112] Yahagi N , Nishizawa T , Akimoto T , Ochiai Y , Goto O . New endoscopic suturing method: String clip suturing method. Gastrointest. Endosc. 2016; 84: 1064–5. 27327846 10.1016/j.gie.2016.05.054

[113] Ye L‐P , Mao X‐L , Zheng H‐H , *et al*. Safety of endoscopic resection for duodenal subepithelial lesions with wound closure using clips and an endoloop: An analysis of 68 cases. Surg. Endosc. 2017; 31: 1070–7. 27387179 10.1007/s00464-016-5065-9

[114] Mori H , Shintaro F , Kobara H , *et al*. Successful closing of duodenal ulcer after endoscopic submucosal dissection with over‐the‐scope clip to prevent delayed perforation. Dig. Endosc. 2013; 25: 459–61. 23368742 10.1111/j.1443-1661.2012.01363.x

[115] Doyama H , Tominaga K , Yoshida N , Takemura K , Yamada S . Endoscopic tissue shielding with polyglycolic acid sheets, fibrin glue and clips to prevent delayed perforation after duodenal endoscopic resection. Dig. Endosc. 2014; 26: 41–5. 24750147 10.1111/den.12253

[116] Takimoto K , Imai Y , Matsuyama K . Endoscopic tissue shielding method with polyglycolic acid sheets and fibrin glue to prevent delayed perforation after duodenal endoscopic submucosal dissection. Dig. Endosc. 2014; 26: 46–9. 24750148 10.1111/den.12280

[117] Tsutsumi K , Kato M , Kakushima N , *et al*. Efficacy of endoscopic preventive procedures to reduce delayed adverse events after endoscopic resection of superficial nonampullary duodenal epithelial tumors: A meta‐analysis of observational comparative trials. Gastrointest. Endosc. 2021; 93: 367–74.e3. 32835670 10.1016/j.gie.2020.08.017

[118] Kato M , Ochiai Y , Fukuhara S , *et al*. Clinical impact of closure of the mucosal defect after duodenal endoscopic submucosal dissection. Gastrointest. Endosc. 2019; 89: 87–93. 30055156 10.1016/j.gie.2018.07.026

